# Supplementary material for: Melting of subducted sediments reconciles geophysical images of subduction zones
Source: Nat Commun. 2021 Feb 26;12:1320. doi: 10.1038/s41467-021-21657-8 (PMC7910547; doi:10.1038/s41467-021-21657-8)
Supplement: Supplementary file 1 — Supplementary Information [file 41467_2021_21657_MOESM1_ESM.pdf]

Supplementary Information for 'Melting of subducted sediments reconciles geophysical images of subduction zones'

Michael Förster and Kate Selway

Nature Communications, 2021

Supplementary Note 1: Melting experiments

Further details of the results of sediment and phlogopite-pyroxenite metasome melting experiments are shown in Supplementary Figure 1, together with the water-saturated sediment solidus curve (black line), the phlogopite stability field (purple line), the antigorite/serpentinite stability field (khaki line and green shading), and ranges of slab-top temperatures from the models of Syracuse et al.[1] (green and red lines).

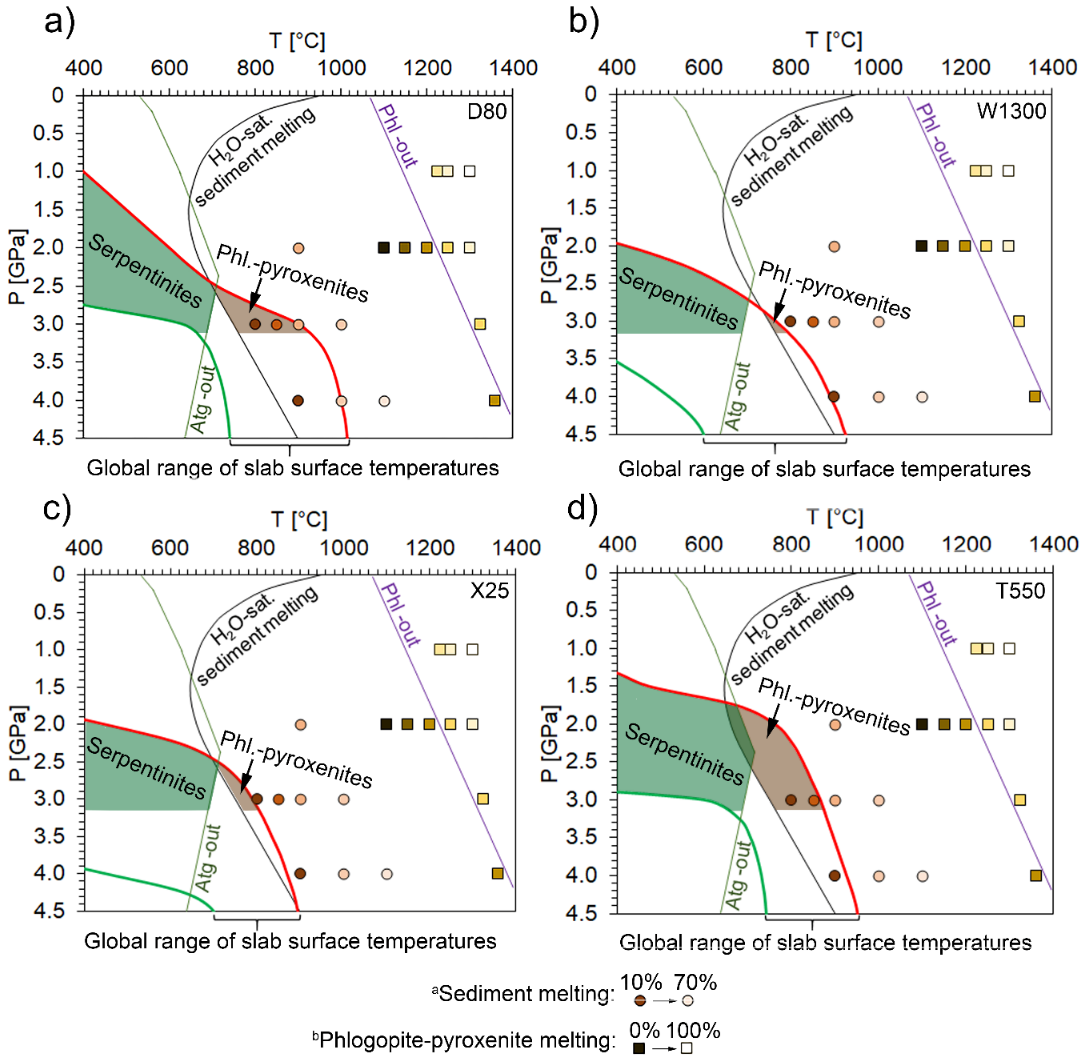

Figure 1: Sediment and phlogopite-pyroxenite melting experiments compared to global slab surface temperatures of the (a) D80, (b) W1300, (c) X25 and (d) T550 model. The global range of slab surface temperatures (area between green and red line) model of Syracuse et al.[1] overlaps with the H_2O saturated sediment melting curve of van Keken et al.[2] within the fore-arc subduction zone mantle. Subducted sediments cross the solidus of H_2O saturated sediment melting at $2.5 - 3.2\,GPa$ and $\sim 675 - 900°C$ to react with mantle peridotites to produce phlogopite pyroxenites (grey shaded area). Reaction experiments confirm sediment melting at fore-arc mantle conditions (circles,[3,4]) and the stability of phlogopite pyroxenites at T of less than $\sim$ 1100°C (squares,[5,6]). All models indicate the formation of phlogopite pyroxenites whereby D80 and T550 show the largest overlap of the temperature gradients with the H_2O-saturated sediment melting curve. At lower pressure and temperatures, serpentinites are present within the stability field of antigorite (green shaded area). Both the serpentinite and phlogopite pyroxenite fields are confined to $> 3.2\,GPa$, which translates to $\sim 100\,km$ and should be regarded as the maximum thickness of the fore-arc lithosphere.

Supplementary Note 2: Synthetic MT modelling

Electrical conductivities were estimated using the compositions and experimental models in Table 1. Multiple components (different minerals, and solid and fluid phases) were combined using a modified Archie's Law[7]. The conductivities of these phases at temperatures typical of the subduction systems being modelled are shown in Supplementary Figure 2, which demonstrates that saline fluids have a stronger conductivity signature than melts, particularly at lower temperatures, and that the phlogopite-pyroxenite metasome phase is highly conductive at higher temperatures.

Name	Composition	References
Peridotite	20% pyroxene, 80% olivine	8, 9
Metasome	20% phlogopite, 80% pyroxene	9, 10
Melt	12% water	11
Saline fluid	5% NaCl	12

Table 1: Compositions and experimental data used to forward model subduction zone electrical resistivities.

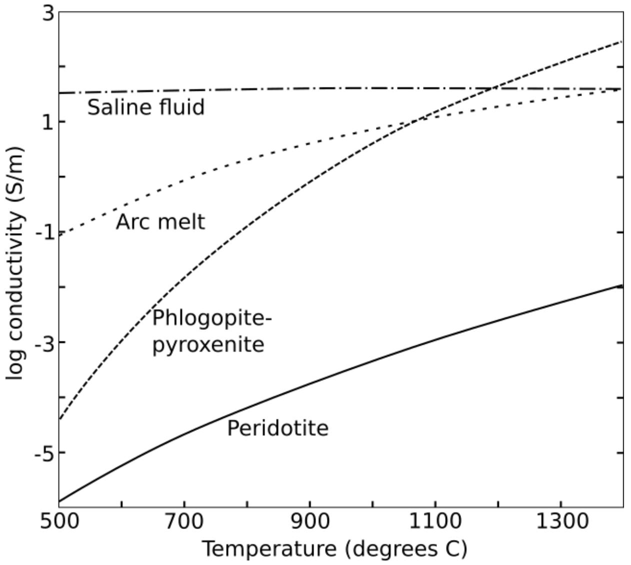

Figure 2: Conductivities of the phases involved in the synthetic MT modelling

By combining these experimental resistivities with the Cascadia and Kyushu modelled thermal structures[1, 2, 13, 14] the predicted electrical resistivity structures of the subduction zones were calculated (Supp. Fig. 3).

Compositions and temperatures in the subduction system are not accurately known. In addition, the conversion of these compositions and temperatures to electrical resistivities requires choosing experimental conductivity models (Table 1) and making assumptions about rock geometry, introducing additional uncertainty into the analysis. In our analysis we make conservative choices for these parameters where possible, using a very simplified compositional model and reliable thermal models. In all regions where fluid or melt is present, modelling was carried out assuming 1% fluid/melt but tests for the Cascadia system were also run with 0.5% and 5% melt/fluid. As shown in Supplementary Figure 4, these different melt proportions altered the magnitude of the conductors but did not strongly affect the overall conductivity structure.

Given that we are modelling a system with strong conductivity contrasts between the melt/fluid phase and the solid phase, the geometric model that is used to calculate total resistivity is an important parameter. The influence of the conductive fluid/melt phase on bulk conductivity will be controlled by its interconnectivity. Supplementary Figure 5 illustrates this point by showing the large contrasts in conductivities between the Hashin-Shtrikman (HS) upper and lower bounds for the peridotite plus

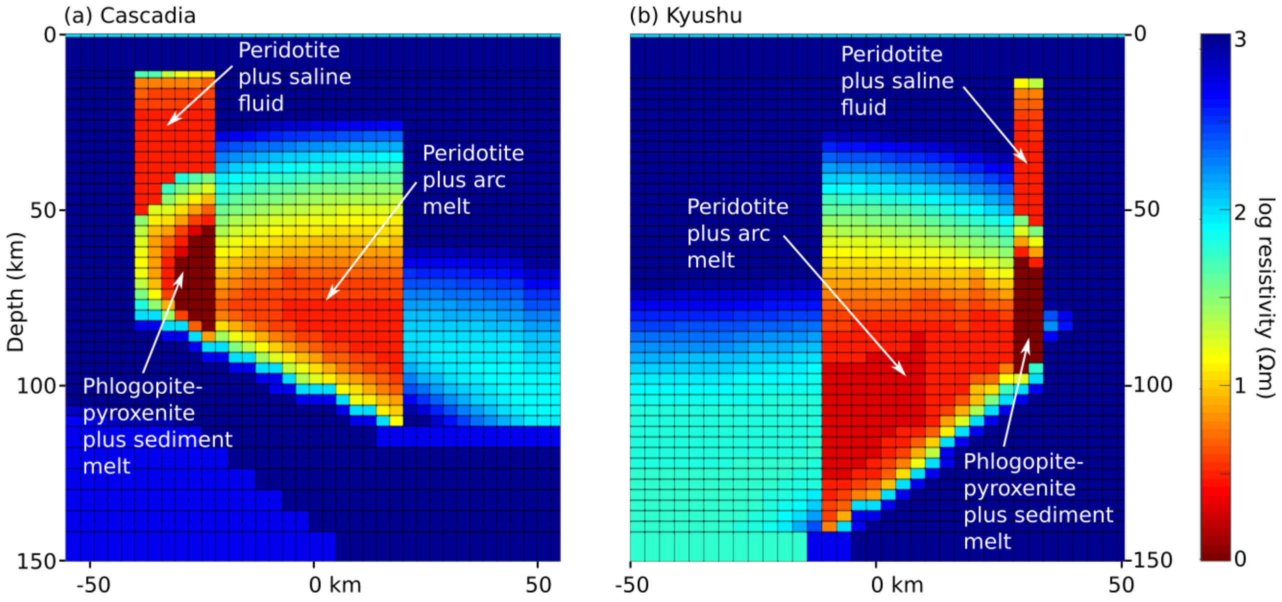

Figure 3: Predicted electrical resistivity structures of the (a) Cascadia and (b) Kyushu subduction systems. The different conductors produced by the regions of modelled phlogopite-pyroxenite metasome with sediment melt, dunite with arc melt, and dunite with saline fluids are marked. Horizontal locations are the same as those in the main text.

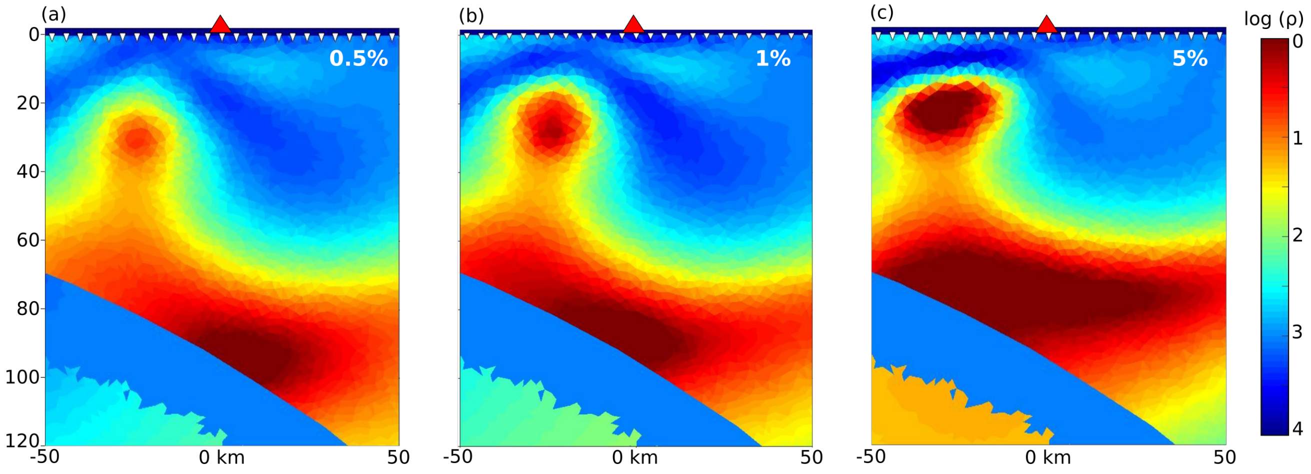

Figure 4: Synthetic models of the Cascadia subduction system with (a) 0.5% and (b) 5% melt and fluid present, compared with 1% shown in the main text.

melt, peridotite plus fluid, and phlogopite-pyroxenite plus melt compositions. The HS upper bound calculates bulk conductivity assuming that the most conductive phase (here the fluid/melt) is perfectly connected, while the lower bound assumes it is disconnected. Experimental and theoretical models suggest that 1% fluid/melt should be well connected[15,16], showing that the actual conductivity is likely to approximate the HS upper bound. Given that the fluid/melt interconnectivity can be estimated, we used a modified Archie's Law[7] to calculate the bulk rock conductivity. We assumed good interconnectivity of the fluid/melt phase, as shown by the close approximation of the Archie's Law conductivity to the HS upper bound. The Cascadia model test run with 0.5% melt/fluid shown in Supplementary Figure 4(a) can also be considered to approximate the conductivity response of a system containing 1% melt/fluid with poorer interconnectivity, showing that the main features are reproduced.

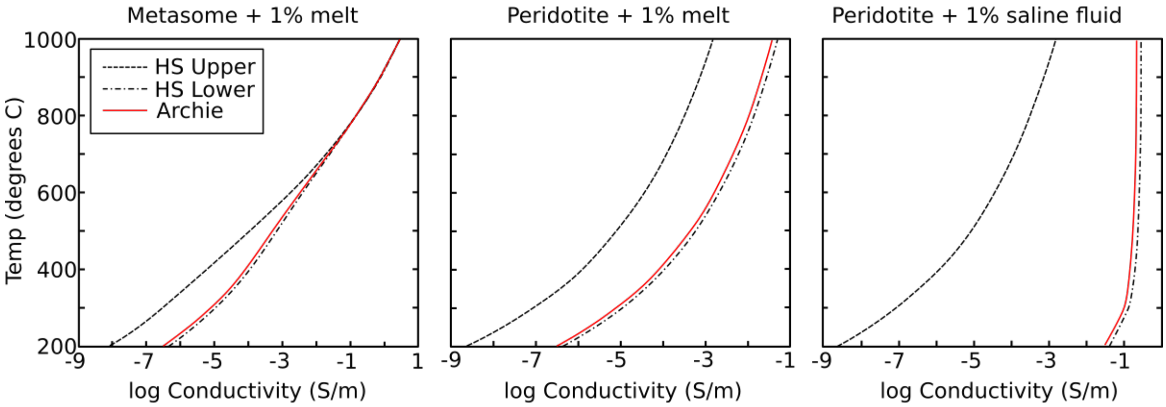

Figure 5: Comparison between bulk conductivities calculated using Hashin-Shtrikman (HS) lower and upper bounds and Archie's Law. As the 1% melt/fluid was assumed to be well connected, the bulk conductivity calculated with Archie's Law approximates the HS upper bound.

3 References

1. Syracuse, E. M., van Keken, P. E. and Abers, G. A. The global range of subduction zone thermal models. Physics of the Earth and Planetary Interiors 183, 73–90; 10.1016/j.pepi.2010.02.004 (2010).

2. van Keken, P. E., Wada, I., Abers, G. A., Hacker, B. R. and Wang, K. Mafic high-pressure rocks are preferentially exhumed from warm subduction settings. Geochem. Geophys. Geosyst. 19, 2934–2961 (2018).

3. Förster, M.W., Prelević, D., Buhre, S., Mertz-Kraus, R., Foley, S.F. An experimental study of the role of partial melts of sediments versus mantle melts in the sources of potassic magmatism. Journal of Asian Earth Sciences 177, 76–88 (2019).

4. Förster, M.W., Foley, S.F., Marschall, H.R., Alard, O., Buhre, S. Melting of sediments in the deep mantle produces saline fluid inclusions in diamonds. Sci. Adv. 5 (5) (2019).

5. Förster, M.W., Prelević, D., Schmück, H.R., Buhre, S., Veter, M., Mertz-Kraus, R., Foley, S.F., Jacob, D.E. Melting and dynamic metasomatism of mixed harzburgite+ glimmerite mantle source: Implications for the genesis of orogenic potassic magmas. Chemical Geology 455, 182–191 (2017).

6. Förster, M.W., Prelević, D., Schmück, H.R., Buhre, S., Marschall, H.R., Mertz-Kraus, R., Jacob, D.E. Melting phlogopite-rich MARID: Lamproites and the role of alkalis in olivine-liquid Ni-partitioning. Chemical Geology 476, 429–440 (2018).

7. Glover, P. W. J. A generalized Archie's law for n phases. Geophysics 75, E247-E265 (2010).

8. Gardès, E., Gaillard, F. and Tarits, P. Toward a unified hydrous olivine electrical conductivity law. Geochem. Geophys. Geosyst. 15, 4984–5000 (2014).

9. Dai, L. and Karato, S.-I. Electrical conductivity of orthopyroxene. Implications for the water content of the asthenosphere. Proceedings of the Japan Academy, Series B 85, 466–475 (2009).

10. Li, Y., Jiang, H. and Yang, X. Fluorine follows water. Effect on electrical conductivity of silicate minerals by experimental constraints from phlogopite. Geochimica et Cosmochimica Acta 217, 16–27 (2017).

11. Sifré, D. et al. Electrical conductivity during incipient melting in the oceanic low-velocity zone. Nature 509, 81–85 (2014).

12. Sinmyo, R. and Keppler, H. Electrical conductivity of NaCl-bearing aqueous fluids to 600°C and 1 GPa. Contrib Mineral Petrol 172, 4 (2017).

13. Abers, G. A., van Keken, P. E. and Hacker, B. R. The cold and relatively dry nature of mantle forearcs in subduction zones. Nature Geosci 10, 333–337 (2017).

14. van Keken, P. E., Hacker, B. R., Syracuse, E. M. and Abers, G. A. Subduction factory. 4. Depth-dependent flux of H2O from subducting slabs worldwide. J. Geophys. Res. Solid Earth 116 (2011).

15. Holtzman, B. Questions on the existence, persistence, and mechanical effects of a very small melt fraction in the asthenosphere. Geochemistry, Geophysics, Geosystems 17 (2), 470-484 (2016)

16. Laumonier, M., Farla, R., Frost, D. J., Katsura, T., Marquardt, K., Bouvier, A. S., Baumgartner, L. P. Experimental determination of melt interconnectivity and electrical conductivity in the upper mantle. Earth and Planetary Science Letters, 463, 286-297 (2017)
